# LH2 Promotes Tumor Progression in Oral Squamous Cell Carcinoma via EPHA7-AKT-VEGF Signaling

**DOI:** 10.7150/jca.141338

**Published:** 2026-09-28

**Authors:** Yuki Nishimura, Atsushi Kasamatsu, Reo Fukushima, Tomoaki Saito, Suguru Okoshi, Isao Miyamoto, Dai Nakashima, Katsuhiro Uzawa

**Affiliations:** 1 Department of Oral Science, Graduate School of Medicine, Chiba University, Chiba, Japan; 2 Department of Dentistry and Oral-Maxillofacial Surgery, Chiba University Hospital, Chiba, Japan

**Keywords:** Lysyl hydroxylase 2, ephrin type-A receptor 7, oral squamous cell carcinoma, cell proliferation, AKT pathway, vascular endothelial growth factor

## Abstract

**Background:**

Lysyl hydroxylase 2 (LH2) specifically hydroxylates lysine residues in type I collagen telopeptides. Aberrant LH2 expression has been implicated as a driver of aggressive phenotypes in multiple cancers, including oral squamous cell carcinoma (OSCC). However, the intracellular signaling mechanisms activated by LH2 overexpression remain unclear.

**Methods:**

In this study, we performed a comprehensive expression analysis to clarify LH2-driven signaling and to identify potential therapeutic targets. Stable LH2-overexpressing (LH2 O/E) OSCC cell lines were generated using a CAG-driven full-length LH2 cDNA construct. Cell proliferation assays were performed to evaluate the effects of LH2 overexpression on cell growth. Expression profiling by RNA sequencing, followed by Ingenuity Pathway Analysis, was performed to identify LH2-regulated networks. Differentially expressed genes were validated by quantitative RT-PCR and immunoblot analyses. siRNA-mediated LH2 knockdown was performed for loss-of-function studies.

**Results:**

LH2 O/E cells displayed significantly increased proliferative capacity compared with control cells. RNA sequencing identified 3,415 differentially expressed genes (1,828 upregulated and 1,587 downregulated) associated with axonal guidance signaling. Among the genes within the axonal guidance signaling network, ephrin type-A receptor 7 (EPHA7) was markedly upregulated. Increased EPHA7 expression was associated with induction of vascular endothelial growth factors (VEGFs) and increased AKT phosphorylation. Knockdown of LH2 in LH2 O/E cells reduced EPHA7 and VEGF levels and attenuated cell proliferation, supporting a functional link between LH2 and the EPHA7-VEGF axis.

**Conclusion:**

Our findings demonstrate that LH2 overexpression drives an EPHA7-VEGF signaling cascade that promotes tumor progression in OSCC. These results provide mechanistic insight into LH2-associated oncogenesis and identify a potential therapeutic target for OSCC.

## Introduction

Oral squamous cell carcinoma (OSCC) is one of the most common malignancies affecting the oral cavity and is characterized by aggressive local invasion and a high rate of cervical lymph node metastasis 1,2. Despite advances in diagnostic modalities, including imaging and molecular marker-based approaches, OSCC is frequently diagnosed at advanced stages, resulting in considerable morbidity and mortality worldwide. OSCC development and progression are driven by a complex interplay of genetic and molecular alterations, including *TP53* mutations, overexpression of the epidermal growth factor receptor, and dysregulation of multiple oncogenic signaling pathways; these alterations collectively promote tumor growth and metastatic dissemination 3,4. Current standard-of-care treatments comprise surgery, radiotherapy, and chemotherapy. Although molecularly targeted therapies and immune checkpoint inhibitors have demonstrated clinical promise, their efficacy remains limited in patients with advanced or metastatic disease 5.

Remodeling of the extracellular matrix (ECM) has emerged as a critical determinant of cancer invasion and metastatic dissemination. Among the ECM modifications associated with tumor progression, aberrant collagen crosslinking is particularly important. Excessive crosslink formation increases matrix stiffness, thereby generating a pro-tumorigenic mechanical microenvironment that facilitates tumor cell migration and invasion 6. In OSCC specifically, abnormal collagen architecture and enhanced collagen crosslink formation have been documented in tumor tissues and are closely associated with aggressive clinical behavior 7.

Lysyl hydroxylase 2 (LH2; encoded by *procollagen-lysine, 2-oxoglutarate 5-dioxygenase 2* [*PLOD2*]) is a collagen-modifying enzyme that hydroxylates specific telopeptidyl lysine residues required for the formation of stable intermolecular collagen crosslinks. Dysregulated LH2 activity contributes to pathological ECM stiffening and has been associated with tumor invasion and metastatic dissemination in various malignancies 8,9. LH2 overexpression has been observed in colorectal cancer, hepatocellular carcinoma, prostate cancer, and head and neck SCC (HNSCC) and is consistently associated with advanced disease stage and poor clinical outcomes 9-13. In OSCC, we previously demonstrated that elevated LH2 expression is correlated with aberrant collagen crosslinking in tumor tissues, suggesting that LH2-mediated ECM remodeling contributes to tumor aggressiveness 7.

However, it remains unclear whether LH2 exerts cell-autonomous effects beyond ECM remodeling and, if so, which downstream molecular mechanisms mediate its role in malignant progression in OSCC. To address this knowledge gap, we generated stable LH2-overexpressing (LH2 O/E) murine OSCC cell lines and performed transcriptomic profiling by RNA sequencing (RNA-seq) to identify downstream molecular targets regulated by LH2. We then investigated the molecular mechanisms by which LH2 promotes malignant phenotypes in OSCC cells.

## Materials and methods

### Cell culture

The mouse OSCC line MOC1 was purchased from Kerafast (Boston, MA, USA) with an authentication certificate 14. MOC1 cells were cultured in a 2:1 mixture of Iscove's Modified Dulbecco's Medium (FUJIFILM Wako, Osaka, Japan) and Ham's Nutrient Mixture F-12 (Cytiva, Buckinghamshire, UK), supplemented with 5% fetal bovine serum (FBS; Sigma-Aldrich, Burlington, MA, USA), 1% penicillin/streptomycin (Sigma-Aldrich), insulin (5 mg/L; Sigma-Aldrich), hydrocortisone (40 µg/L; Sigma-Aldrich), and epidermal growth factor (5 µg/L; Sigma-Aldrich). The mouse OSCC cell line Sq1979 was obtained from the RIKEN BioResource Center (Tsukuba, Ibaraki, Japan) with an authentication certificate 15. Sq1979 cells were cultured in Eagle's Minimum Essential Medium (Sigma-Aldrich) supplemented with 10% FBS and 1% penicillin/streptomycin. Both cell lines were maintained in a humidified incubator containing 5% CO₂ at 37 °C, and the culture media were replaced twice per week.

### Establishment of LH2 O/E cell lines

Cells were transfected with plasmid DNA using Lipofectamine 2000 (Thermo Fisher Scientific, Waltham, MA, USA). The overexpression construct pRP[Exp]-Neo-CAG>mPlod2, generated by VectorBuilder Inc. (Chicago, IL, USA), was used to establish LH2 O/E cells. A corresponding control vector, pRP[Exp]-Neo-CAG, was used to generate mock cells. For MOC1 cells, transfection was performed in 6-well plates using 14 μg plasmid DNA and 6.0 μL Lipofectamine 2000 per well. For Sq1979 cells, transfection was performed in 6-well plates using 14 μg plasmid DNA and 4.5 μL Lipofectamine 2000 per well. Following transfection, both cell lines were subjected to selection using G418 (250 µg/mL; Thermo Fisher Scientific), and resistant colonies were isolated by colony picking. For each cell line, three independent LH2 O/E clones and three mock clones were established. Expanded clones were used for subsequent experiments.

### Quantitative reverse-transcription PCR (qRT-PCR) analysis

Total RNA was extracted from stable LH2 O/E and mock clones, derived from both MOC1 and Sq1979 cells, using TRIzol reagent (Thermo Fisher Scientific). First-strand cDNA was synthesized using ReverTra Ace® qPCR RT Master Mix (TOYOBO, Osaka, Japan). qRT-PCR was performed using TaqMan™ Fast Advanced Master Mix and TaqMan Gene Expression Assays (Thermo Fisher Scientific) on the LightCycler 96 System (Roche Diagnostics, Basel, Switzerland). The following TaqMan Gene Expression Assays were used according to the manufacturer's instructions: *Plod2* (LH2; Mm00478767_m1), ephrin type-A receptor 7 (*Epha7*; Mm01217450_m1), vascular endothelial growth factor (*Vegf)-B* (Mm00442102_m1), *Vegf-C* (Mm00437310_m1), and *Gapdh* (Mm99999915_g1), which served as the endogenous control. Relative mRNA expression levels were quantified using standard curves, generated from the expression levels of serial dilutions of cDNA, and normalized to *Gapdh*. Each independent biological replicate (n = 3) was analyzed in technical triplicate.

### Immunoblot analysis

Cells were harvested and lysed in urea lysis buffer (7 M urea, 2 M thiourea, 4% w/v CHAPS, 10 mM Tris, pH 7.4) supplemented with a protease inhibitor cocktail (Roche Diagnostics). Protein concentrations were determined using the Bradford assay. Samples containing equal amounts of protein (20 µg) were mixed with reducing sample buffer containing β-mercaptoethanol (FUJIFILM Wako), denatured at 95 °C for 5 min, and separated under denaturing and reducing conditions on 4-12% NuPAGE Bis-Tris precast gels (Thermo Fisher Scientific) at 150 V for 1 h. Proteins were then transferred onto PVDF membranes at 50 V for 75 min. Membranes were blocked with Blocking One (Nacalai Tesque, Kyoto, Japan) for 1 h at room temperature and subsequently incubated overnight at 4 °C with the following primary antibodies: rabbit anti-mouse LH2 (1:1000; 21214-1-AP, Proteintech, Rosemont, IL, USA), mouse monoclonal anti-EPHA7 (1:500; 66667-1-Ig, Proteintech), rabbit anti-human AKT (mouse cross-reactive) (1:1000; 10176-2-AP, Proteintech), rabbit anti-human phospho-AKT (Ser473) (mouse cross-reactive) (1:3000; 28731-1-AP, Proteintech), and mouse anti-GAPDH (1:500; sc-32233, Santa Cruz Biotechnology, Dallas, TX, USA). After washing three times with TBS-T (0.1% Tween-20), the membranes were incubated with HRP-conjugated anti-rabbit or anti-mouse IgG secondary antibody (Promega, Madison, WI, USA), according to the manufacturer's instructions. Protein bands were visualized using ECL Prime Western Blotting Detection Reagent (Cytiva) and imaged using the ChemiDoc XRS Plus system (Bio-Rad Laboratories, Hercules, CA, USA). Band intensities were quantified using Image Lab software (Bio-Rad Laboratories). Phospho-AKT levels were normalized to total AKT levels, whereas the other target protein levels were normalized to GAPDH. Biological replicates comprised independent samples (n = 3).

### Cell proliferation assay

Cell proliferation was evaluated using the CellTiter 96® AQueous One Solution Cell Proliferation Assay (MTS; Promega) according to the manufacturer's instructions. Cells were seeded into 96-well plates at 1.5 × 10^3^/well in 100 µL culture medium. Three independent mock clones and three independent LH2 O/E clones were analyzed, with each clone plated in triplicate wells. Cell proliferation was assessed at 24, 48, 72, and 96 h after cell seeding. Briefly, 20 µL MTS reagent was added to each well, followed by incubation for 1 h at 37 °C in a humidified atmosphere containing 5% CO₂. Absorbance was measured at 490 nm using the Benchmark Plus Microplate Reader (Bio-Rad Laboratories).

### RNA-seq and bioinformatic analysis

Total RNA was isolated from three independent LH2 O/E and mock clones each using TRIzol reagent (Thermo Fisher Scientific). RNA integrity was assessed using a microfluidics-based quality evaluation system; all samples that exhibited an RNA integrity number ≥ 9.4 were used for downstream analyses. Ribosomal RNA depletion and strand-specific library preparation were performed using the MGIEasy rRNA Depletion Kit (v1.3) and the MGIEasy Fast RNA Library Prep Set (both from MGI Tech, Shenzhen, China), respectively. Libraries were sequenced on the MGI DNBSEQ-G400 FAST platform with paired-end 150 bp reads. Raw sequencing reads were quality-trimmed using Trimmomatic (v0.38). Trimmed reads were aligned to the mouse reference genome (GRCm39) using HISAT2 (v2.1.0), and gene-level expression was estimated from the resulting alignments using RSEM (v1.3.0). Differential gene expression analysis was conducted using the edgeR package, with expression levels normalized to counts per million. Genes with an absolute log2 fold change (|log2FC|) ≥ 1 and nominal P-value ≤ 0.05 were defined as differentially expressed genes (DEGs). Volcano plots were generated from log2FC and -log10(P-value) values, with significantly upregulated and downregulated genes highlighted in red and blue, respectively. Gene Ontology (GO) enrichment analysis of the significantly upregulated DEGs was performed using the Biological Process (BP) ontology in QIAGEN CLC Genomics Workbench (QIAGEN, Hilden, Germany). Canonical pathway analysis of the genes meeting the criteria |log2FC| ≥ 1 and nominal P-value ≤ 0.05 was conducted using Ingenuity Pathway Analysis (IPA; QIAGEN) to identify the signaling pathways significantly associated with LH2 overexpression.

### Enzyme-linked immunosorbent assay (ELISA)

ELISA for VEGF-B and VEGF-C was performed using culture supernatants obtained from MOC1 and Sq1979 transfected with either the LH2 O/E or mock construct. For both cell lines, cells were seeded in 6-well plates at 5.0 × 10^5^/well. After cell attachment, the medium was replaced with corresponding serum-free culture medium for 48 h, after which the culture media were collected. The collected media were centrifuged at 500 × g for 10 min to remove cellular debris, and the clarified supernatants were subjected to ELISA. The VEGF-B and VEGF-C concentrations were measured using the Mouse VEGF-B ELISA Kit (ab289700, Abcam, Cambridge, UK) and pan-HMR VEGF-C ELISA Kit (ab315050, Abcam), respectively, following the manufacturer's instructions. Absorbance was recorded at 450 nm using the Benchmark Plus Microplate Reader (Bio-Rad Laboratories). Three biological replicates were analyzed for each condition (n = 3).

### Gene knockdown

Small interfering RNA (siRNA)-mediated knockdown of *Plod2*, which encodes LH2, was performed in LH2 O/E clones derived from MOC1 and Sq1979 cells. Stealth RNAi™ siRNAs targeting mouse *Plod2* (Oligo IDs: MSS218582, MSS218583, and MSS218584; Thermo Fisher Scientific) were used for *Plod2* knockdown, and the efficacy of each siRNA was evaluated independently. Stealth RNAi™ siRNA Negative Control LO GC (12935-200; Thermo Fisher Scientific) was used as a non-targeting control. Cells were seeded into 6-well plates at 5.0 × 10⁵/well and transfected using Lipofectamine™ RNAiMAX Transfection Reagent (Thermo Fisher Scientific) according to the manufacturer's instructions. siRNA-lipid complexes were prepared in Opti-MEM® medium, and 30 pmol siRNA was added to each well. Total RNA was extracted at 24 h and 48 h after siRNA transfection. The knockdown efficiency of *Plod2* was confirmed by qRT-PCR as described above.

For cell proliferation assays, cells were seeded into 96-well plates at 1.5 × 10³/well in 100 µL culture medium. siRNA transfection was performed at 24 h after cell seeding using Lipofectamine™ RNAiMAX under optimized conditions. Cell proliferation was assessed at 24, 48, 72, and 96 h after seeding of the transfected cells using the MTS assay as described above. Experiments were performed using three biological replicates for each condition (n = 3).

### The Cancer Genome Atlas (TCGA) data acquisition and processing

RNA-seq expression data for HNSCC were obtained from FireBrowse (http://firebrowse.org). Anatomic subsite information was obtained from the Genomic Data Commons (GDC) Data Portal (https://portal.gdc.cancer.gov/), and clinical information, including age, TNM classification, pathological stage, and survival outcome, was obtained from cBioPortal (http://www.cbioportal.org). A total of 331 patients with HNSCC and available overall survival (OS) data were included in the survival analysis. The cohort included tumors from multiple head and neck subsites, predominantly arising from the oral cavity. Expression values for *PLOD2* and *EPHA7* were log2-transformed prior to analysis.

### Survival analysis

OS was evaluated using the Kaplan-Meier method, and survival distributions between high- and low-expression groups were compared using the log-rank test. Univariate Cox proportional hazards regression analyses were performed to estimate hazard ratios (HRs) and 95% confidence intervals (CIs) for *PLOD2* and *EPHA7* individually (n = 331 for each analysis) and for the combined *PLOD2*-high/*EPHA7*-high versus *PLOD2*-low/*EPHA7*-low comparison (n = 95). 5-year OS was assessed with follow-up censored at 60 months. All statistical analyses were performed using JMP software (SAS Institute Inc., Cary, NC, USA). Kaplan-Meier curves were re-plotted using R software for graphical presentation.

### Statistical analysis

Statistical differences were analyzed using Student's t-test, and *P* values < 0.05 were considered statistically significant. All experiments were independently performed in triplicate. Data are expressed as the mean ± standard deviation (SD).

## Results

### Establishment and functional characterization of LH2 O/E OSCC cells

To investigate the functional role of LH2 in OSCC cells, we established LH2 O/E OSCC cells from MOC1 and Sq1979 cell lines, together with their corresponding mock cells. To confirm overexpression of LH2, qRT-PCR and immunoblot analyses were performed. qRT-PCR and immunoblot analyses both demonstrated that LH2 mRNA and protein levels, respectively, were significantly (*P* < 0.001) increased in LH2 O/E cells compared with mock cells (Fig. 1A, B, D, E), demonstrating successful establishment of LH2 O/E OSCC cell lines.

To examine the effect of LH2 overexpression on cell proliferation, cell growth was monitored for up to 96 h after seeding. In both MOC1 and Sq1979 cells, LH2 overexpression significantly enhanced proliferation compared with mock cells, with significant (*P* < 0.05, 0.01, or 0.001) differences observed at 72 and 96 h (Fig. 1C, F). These findings indicate that LH2 overexpression promotes cell proliferation in OSCC cells.

### Gene expression changes induced by LH2 overexpression in OSCC cells

To characterize the transcriptional changes induced by LH2 overexpression, RNA-seq analysis was performed in LH2 O/E and mock OSCC cells derived from the MOC1 cell line. Of the 21,009 genes analyzed, 3,415 were identified as significantly differentially expressed based on the criteria of |log2FC| ≥ 1 and nominal P-value ≤ 0.05. Of these DEGs, 1,828 were significantly upregulated and 1,587 significantly downregulated in LH2 O/E cells compared with mock cells (Fig. 2A). We next focused on the significantly upregulated genes and performed pathway enrichment analysis. This analysis revealed that the axonal guidance signaling pathway is the most significantly altered pathway in LH2 O/E cells (Fig. 2B). To further explore the relationship between *Plod2* and axonal guidance genes, gene network analysis was conducted using IPA. Among the 112 upregulated genes involved in the axonal guidance signaling pathway, *Epha7* was the only gene directly connected to *Plod2* in the generated interaction network (Fig. 2C). These findings suggest that LH2 overexpression is associated with broad transcriptional reprogramming, and *Plod2* may exert its downstream effects, at least in part, via modulation of the axonal guidance signaling pathway and *Epha7*-related signaling.

### Upregulation of EPHA7 in LH2 O/E OSCC cells

Based on the RNA-seq and pathway analyses, we next examined EPHA7 expression in LH2 O/E OSCC cells. To evaluate EPHA7 mRNA and protein levels, qRT-PCR and immunoblot analyses, respectively, were performed in LH2 O/E and mock cells in both MOC1 and Sq1979 cells (Fig. 3A, B, C, D). qRT-PCR analysis demonstrated that *Epha7* mRNA expression was significantly (*P* < 0.001) increased in LH2 O/E cells compared with mock cells (Fig. 3A, C). Consistently, immunoblot analysis revealed that EPHA7 protein levels were significantly (*P* < 0.001) elevated in LH2 O/E cells relative to mock cells (Fig. 3B, D). Thus, EPHA7 expression was upregulated at both the mRNA and protein levels in LH2 O/E OSCC cells.

### Upregulation of VEGF-B and VEGF-C in LH2 O/E OSCC cells

Immunoblot analyses demonstrated that phosphorylation of AKT was significantly (*P* < 0.001) increased in LH2 O/E cells compared with mock cells, suggesting that activation of AKT signaling is associated with LH2 overexpression (Fig. 4A). To determine a potential effect of LH2 overexpression on the expression of cell proliferation-related genes, we examined the mRNA expression levels of *Vegf-B* and *Vegf-C* by qRT-PCR. Both *Vegf-B* and *Vegf-C* mRNA levels were significantly increased in LH2 O/E cells relative to mock cells for both MOC1 and Sq1979 cells (*P* < 0.001) (Fig. 4B, D). These results suggest that Vegf-B and Vegf-C may function downstream of the LH2-EPHA7 signaling axis. To determine whether the increased gene expression was associated with enhanced protein secretion, the VEGF-B and VEGF-C concentrations in conditioned media were quantified by ELISA. The secreted levels of both VEGF-B and VEGF-C were significantly elevated in LH2 O/E cells relative to mock cells (*P* < 0.001) (Fig. 4C, E). Therefore, LH2 overexpression resulted in both increased expression and secretion of VEGF-B and VEGF-C in OSCC cells.

### Effect of *Plod2* knockdown on *Epha7* expression

To further examine the relationship between *Plod2* and *Epha7* expression, *Plod2* was knocked down in LH2 O/E OSCC cells. Cells were transfected with *Plod2*-specific siRNA (si*Plod2*) or a negative control siRNA (si*Control*), and the efficiency of *Plod2* knockdown was confirmed by qRT-PCR. *Plod2* mRNA expression was significantly (*P* < 0.001) reduced in si*Plod2*-treated LH2 O/E cells compared with si*Control-*treated LH2 O/E cells in both MOC1 and Sq1979 cell lines (Fig. 5A, F). qRT-PCR analysis further demonstrated that *Epha7* mRNA expression was significantly (*P* < 0.001) decreased in si*Plod2*-treated LH2 O/E cells compared with siControl cells in both cell lines (Fig. 5B, G). Consistent with these findings, immunoblot analysis demonstrated that EPHA7 protein expression was significantly reduced following *Plod2* knockdown in both LH2 O/E MOC1 and Sq1979 cells (Supplementary Fig. S1). In addition, *Vegf-B* and *Vegf-C* mRNA expression levels were also significantly (*P* < 0.001) reduced following *Plod2* knockdown (Fig. 5C, D, H, I).

Next, the effect of *Plod2* knockdown on cell proliferation after transfection with si*Plod2* or si*Control* was assessed. The proliferation of si*Plod2*-treated LH2 O/E cells in both MOC1 and Sq1979 cell lines was significantly decreased at 72 h (*P* < 0.05) and further reduced at 96 h (*P* < 0.01) compared with si*Control* cells (Fig. 5E, J). Altogether, we found that *Plod2* knockdown was associated with reduced *Epha7*, *Vegf-B*, and *Vegf-C* expression and suppressed proliferation in OSCC cells.

### Clinical relevance of *PLOD2* and *EPHA7* expression in the TCGA cohort

To assess the clinical relevance of *PLOD2* and *EPHA7*, we performed survival analyses using the TCGA cohort. Patients with high *PLOD2* expression showed significantly poorer 5-year OS than those with low* PLOD2* expression (Fig. 6A). Similarly, patients with high *EPHA7* expression showed significantly poorer 5-year OS than those with low *EPHA7* expression (Fig. 6B). Furthermore, patients with high expression of both *PLOD2* and *EPHA7* showed significantly poorer 5-year OS than those with low expression of both genes (Fig. 6C).

## Discussion

LH2, encoded by *Plod2*, is a collagen-modifying enzyme that catalyzes hydroxylation of lysine residues in the telopeptidyl domains of fibrillar collagens, thereby facilitating the formation of stable intermolecular collagen crosslinks and regulating ECM organization 16,17. Initially investigated in the context of connective tissue biology and collagen-related disorders such as Bruck syndrome 18,19, LH2 is increasingly being recognized as an important regulator of malignant progression in multiple cancers, including OSCC 7-9,11,12,20. In OSCC, LH2-mediated collagen crosslinking contributes to the formation of a mechanically permissive tumor microenvironment that facilitates cancer cell migration, invasion, and metastasis 7,20. Moreover, recent studies have demonstrated that LH2 exerts functions other than ECM remodeling, including regulation of cancer cell plasticity, stemness, and intracellular signaling pathways 11,12. However, the molecular mechanisms linking LH2 overexpression to malignant phenotypes in OSCC remain incompletely understood. In the present study, we demonstrated that LH2 overexpression significantly enhanced OSCC cell proliferation and induced widespread transcriptional alterations. RNA-seq identified the axonal guidance signaling pathway as the most significantly enriched pathway among the affected genes, and subsequent analyses revealed that LH2 overexpression was associated with increased EPHA7 expression, AKT phosphorylation, and VEGF-B/VEGF-C expression and secretion. Collectively, these findings suggest a previously unrecognized signaling axis linking LH2 to angiogenic and lymphangiogenic pathways in OSCC.

Previous studies, including those from our group, have focused primarily on the role of LH2 in ECM remodeling via hydroxylation-dependent collagen crosslinking 7,20. In HNSCCs, LH2 has been shown to promote invasion and metastasis via hydroxylation-dependent functional activation of integrin β1 independently of epithelial-mesenchymal transition, suggesting that LH2 influences tumor progression through mechanisms other than simple ECM stiffening 20. In addition, a recent study demonstrated that the LH2-succinate axis regulates cancer cell stemness and epithelial-mesenchymal plasticity, further supporting the concept that LH2 exerts broader biological effects outside of extracellular collagen modification 11. Consistent with these observations, our RNA-seq analysis identified 3,415 significant DEGs in LH2 O/E OSCC cells, indicating that LH2 overexpression induces substantial transcriptional reprogramming. These findings suggest that LH2 possesses cell-autonomous oncogenic functions in OSCC cells in addition to its canonical role in ECM remodeling.

LH2 is primarily localized to the endoplasmic reticulum and does not function as a transcription factor. Therefore, the increase in EPHA7 expression observed in LH2 O/E cells is unlikely to reflect direct transcriptional regulation by LH2. Rather, LH2 overexpression may indirectly influence transcriptional programs through alterations in protein modification and subsequent intracellular or extracellular signaling events, thereby contributing to EPHA7 upregulation. The precise molecular mechanism linking LH2 activity to EPHA7 expression remains to be elucidated.

Among the pathways enriched in LH2 O/E cells, axonal guidance signaling was identified as the most significantly altered pathway. Axonal guidance molecules—including Eph receptors and ephrin ligands, semaphorins, netrins, and slits—were originally characterized as regulators of neural circuit formation and tissue patterning during development but are now recognized as important mediators of cancer progression 21,22. In OSCC, the axonal guidance molecule semaphorin 7A has been reported to promote tumor growth and metastasis by regulating G1 cell cycle progression and matrix metalloproteases, with possible contribution to tumoral angiogenesis 23. This further illustrates that axonal guidance molecules are functionally repurposed during oral carcinogenesis to support multiple aspects of malignant progression. Eph receptors constitute the largest family of receptor tyrosine kinases and exhibit context-dependent functions in cancer, acting either as tumor suppressors or tumor promoters depending on receptor subtype, ligand availability, and cellular context 24-28. In the present study, EPHA7 was identified by IPA as the only gene within the axonal guidance signaling pathway directly linked to *Plod2*, and its upregulation was confirmed at both the mRNA and protein levels in LH2 O/E OSCC cells. Furthermore, *Plod2* knockdown reduced *Epha7* expression, supporting a functional relationship between LH2 and EPHA7 expression. Although EPHA7 has been reported as a soluble tumor suppressor in follicular lymphoma 29, studies of its role in epithelial malignancies remain limited. Notably, EPHA7 silencing has been shown to suppress proliferation, migration, and invasion in laryngeal SCC line AMC-HN-8 cells 30, suggesting that the biological role of EPHA7 may be highly context dependent and, in certain epithelial cancers, tumor-promoting. To our knowledge, the present study provides the first evidence of an association between LH2 and EPHA7 in OSCC.

We further observed significantly increased phosphorylation of AKT in LH2 O/E OSCC cells. Dysregulation of the PI3K/AKT pathway is one of the most frequent oncogenic events in OSCC and contributes to tumor cell proliferation, survival, and therapeutic resistance 31. AKT activation in OSCC is commonly induced downstream of receptor tyrosine kinase signaling, and Eph receptor signaling has been implicated in regulating the PI3K/AKT pathway in multiple cancer types 32. Therefore, the enhanced AKT phosphorylation observed in LH2 O/E cells may be mediated, at least in part, by LH2-induced EPHA7 upregulation.

Another important finding of the present study was the increased expression and secretion of VEGF-B and VEGF-C in LH2 O/E OSCC cells. VEGF-B has been implicated in tumor progression and metastatic dissemination in several malignancies 33, whereas VEGF-C is a key mediator of lymphangiogenesis via VEGFR-3 signaling and plays a critical role in lymph node metastasis 34. Cervical lymph node metastasis is one of the strongest prognostic determinants in OSCC and is associated with markedly reduced survival outcomes 35,36. Therefore, the finding that LH2 overexpression promoted VEGF-C secretion may have important clinical implications. In HNSCCs, elevated VEGF-C expression has been associated with lymphangiogenesis and lymph node metastasis 37. Furthermore, AKT signaling is a well-established regulator of VEGF expression and angiogenic responses 38, providing a plausible mechanism by which LH2-induced EPHA7/AKT activation may contribute to the observed upregulation of VEGF-B and VEGF-C in this study. Given that *Plod2* knockdown concurrently reduced *Vegf-B* and *Vegf-C* expression, our findings support a model in which LH2-induced EPHA7 upregulation promotes AKT activation, thereby contributing to VEGF-B and VEGF-C induction. Collectively, these observations suggest that LH2 may promote OSCC progression through complementary mechanisms: canonical ECM remodeling via aberrant collagen crosslinking and non-canonical activation of intracellular signaling pathways associated with angiogenesis and lymphangiogenesis.

A limitation of this study is that our findings were obtained using murine OSCC cell lines under *in vitro* conditions. Although the functional LH2-EPHA7-AKT-VEGF signaling axis remains to be validated in human HNSCC and *in vivo*, our additional analysis of the TCGA cohort provided clinical support for the relevance of *PLOD2* and *EPHA7*. Patients with high *PLOD2* expression and those with high *EPHA7* expression showed significantly poorer 5-year OS than patients with low expression. Furthermore, patients with high expression of both* PLOD2* and *EPHA7* showed significantly poorer 5-year OS than those with low expression of both genes. In addition, the precise mechanism by which LH2 regulates EPHA7 expression, as well as the direct contribution of EPHA7 to AKT activation, was not fully delineated in this study. Although the observed changes in EPHA7 expression following LH2 overexpression and *Plod2* knockdown support a functional association between LH2 and EPHA7, the specific functional contribution of EPHA7 was not directly examined in the present study. *Epha7*-specific knockdown or direct inhibition will therefore be an important focus of future studies to determine whether EPHA7 directly contributes to LH2-associated AKT activation, VEGF induction, and enhanced cell proliferation. Further validation in human OSCC specimens and *in vivo* models will be necessary to establish the biological and clinical significance of this signaling pathway.

In conclusion, the present study demonstrates that LH2 overexpression upregulates EPHA7 expression, AKT phosphorylation, and VEGF-B/VEGF-C expression and secretion in OSCC cells (Fig. 7). These findings support a previously unrecognized association between LH2 and EPHA7-AKT-VEGF signaling and suggest that the oncogenic role of LH2 in OSCC may extend beyond ECM remodeling to include modulation of intracellular signaling pathways associated with angiogenesis and lymphangiogenesis. Further studies directly defining the functional contribution of EPHA7 and validating these findings in human OSCC and *in vivo* models may provide a basis for the development of novel therapeutic strategies targeting LH2-associated signaling in OSCC.

## Supplementary Material

Supplementary Figure S1.

## Figures and Tables

**Figure 1:**
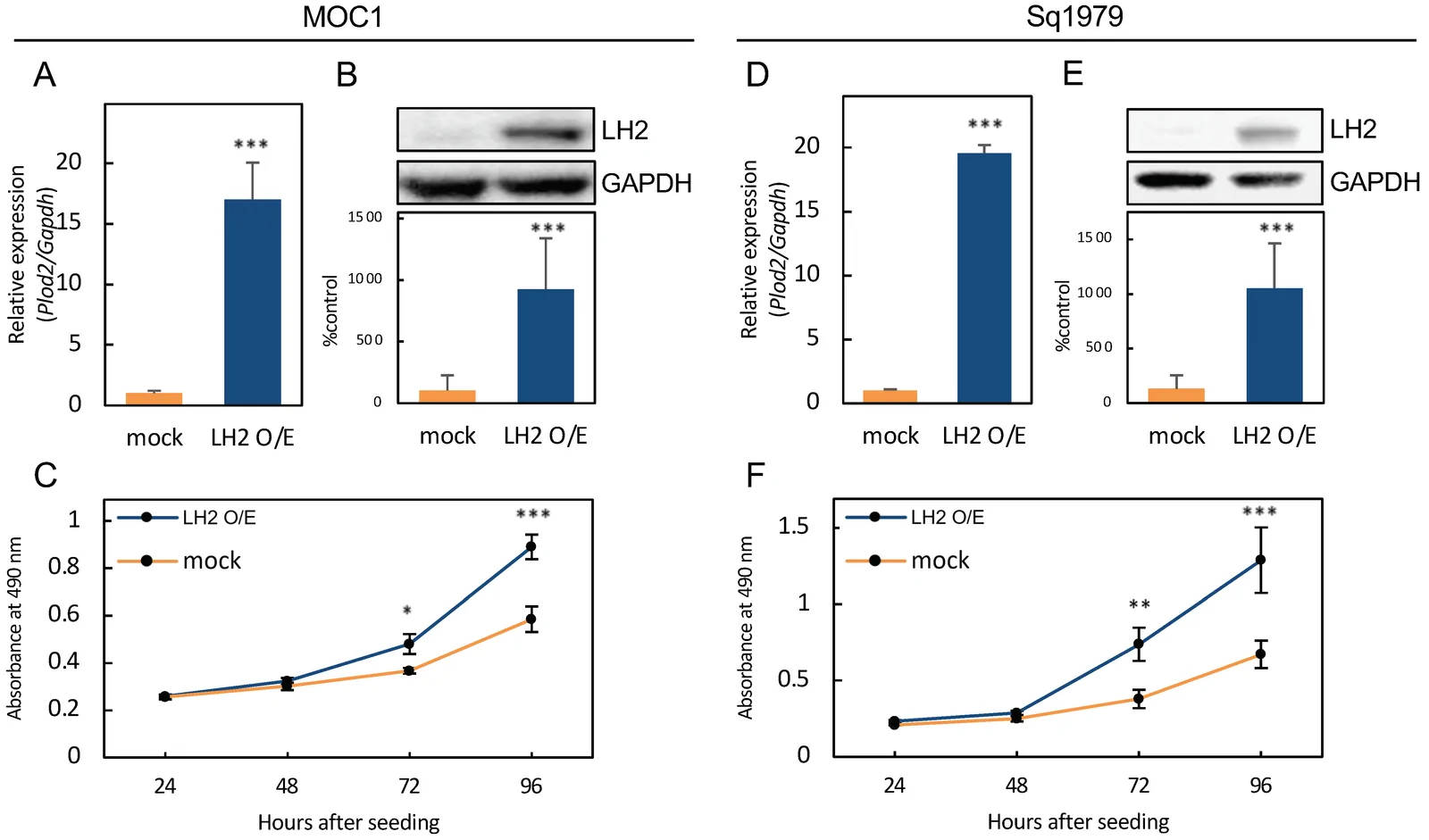
** Establishment and functional characterization of LH2 O/E OSCC cells.** (A, D) qRT-PCR analysis of *Plod2* (LH2) mRNA expression in LH2 O/E and mock cells (MOC1 and Sq1979 cell lines). Relative *Plod2* mRNA levels normalized to *Gapdh* are shown. *Plod2* expression was significantly increased in LH2 O/E cells compared with Mock cells (n = 3 per group; ***P < 0.001; Student's t-test). (B, E) Immunoblot analysis confirming increased LH2 protein expression in LH2 O/E cells compared with mock cells (MOC1 and Sq1979 cell lines) (n = 3 per group; ***P < 0.001; Student's t-test). (C, F) MTS assay showing increased cell proliferation in LH2 O/E cells compared with mock cells (MOC1 and Sq1979 cell lines). LH2 O/E cells exhibited significantly increased proliferation at 72 h and 96 h (*P < 0.05, **P < 0.01, ***P < 0.001). Data are presented as the mean ± SD from three independent experiments.

**Figure 2:**
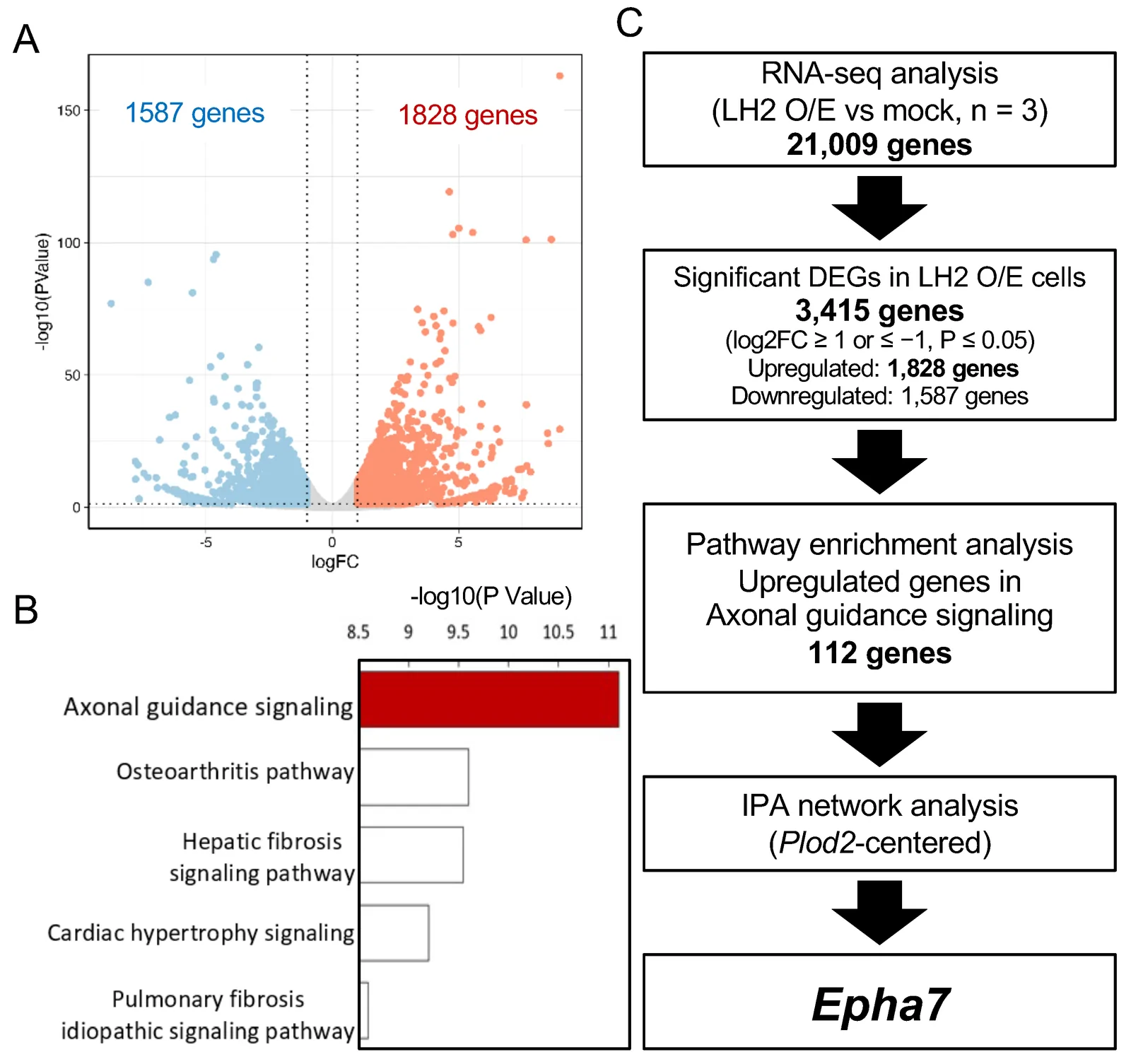
** Transcriptomic changes induced by LH2 overexpression in OSCC cells.** (A) Volcano plot showing the DEGs identified by RNA-seq in LH2 O/E versus mock cells. Genes with |log2FC| ≥ 1 and nominal P-value ≤ 0.05 were defined as DEGs. (B) Pathway enrichment analysis of the significantly upregulated genes identified by RNA-seq. Axonal guidance signaling was the top enriched pathway. (C) Schematic flowchart illustrating the analytical strategy used to identify candidate genes associated with LH2 overexpression. Among the 3,415 significant DEGs, the 1,828 upregulated genes were subjected to pathway enrichment analysis. Of these upregulated genes, 112 were involved in the axonal guidance signaling pathway, among which *Epha7* was the only gene directly connected to *Plod2* in the generated IPA interaction network.

**Figure 3:**
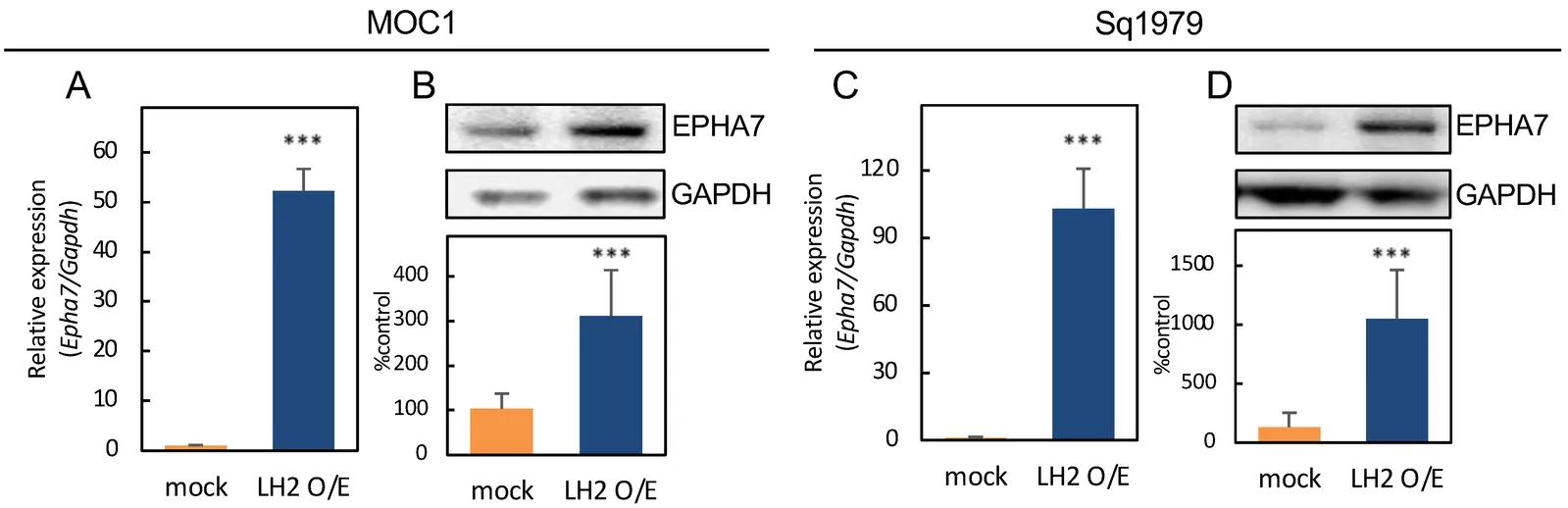
** Upregulation of EPHA7 in LH2 O/E OSCC cells.** (A, C) qRT-PCR analysis of *Epha7* mRNA expression in LH2 O/E and mock cells (MOC1 and Sq1979 cell lines). Relative *Epha7* mRNA levels normalized to *Gapdh* are shown. *Epha7* expression was significantly increased in LH2 O/E cells compared with mock cells (n = 3 per group; ***P < 0.001; Student's t-test). (B, D) Immunoblot analysis confirming increased EPHA7 protein expression in LH2 O/E cells compared with mock cells (MOC1 and Sq1979 cell lines) (n = 3 per group; ***P < 0.001; Student's t-test).

**Figure 4:**
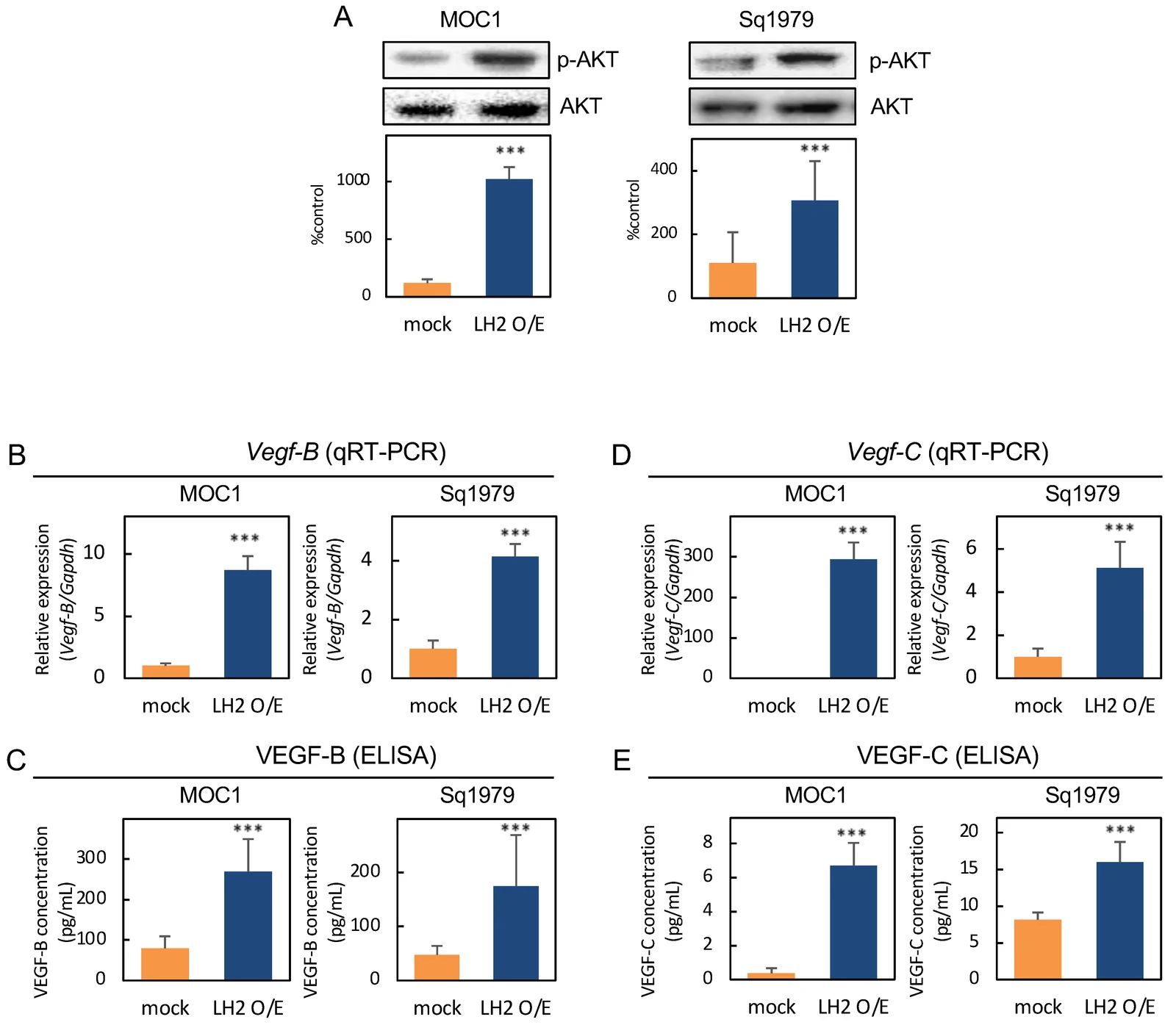
** Upregulation of VEGF-B and VEGF-C following EPHA7 upregulation in LH2 O/E cells.** (A) Immunoblot analysis of AKT signaling in LH2 O/E cells. Increased phosphorylation of AKT (p-AKT) was observed in LH2 O/E cells compared with mock cells (n = 3 per group; ***P < 0.001; Student's t-test). Total AKT was used as a loading control. Each experiment was independently repeated three times. (B, D) qRT-PCR analysis of *Vegf-B* and *Vegf-C* mRNA expression in LH2 O/E and mock cells (MOC1 and Sq1979 cell lines). Relative mRNA expression levels normalized to *Gapdh* are shown and demonstrate significant upregulation of *Vegf-B* and *Vegf-C* in the LH2 O/E cells (n = 3 per group; ***P < 0.001; Student's t-test). (C, E) ELISA of VEGF-B and VEGF-C concentrations in conditioned media from LH2 O/E and mock cells. The secreted levels of VEGF-B and VEGF-C were significantly increased in LH2 O/E cells compared with mock cells (n = 3 per group; ***P < 0.001; Student's t-test). Data are presented as the mean ± SD from three independent experiments.

**Figure 5:**
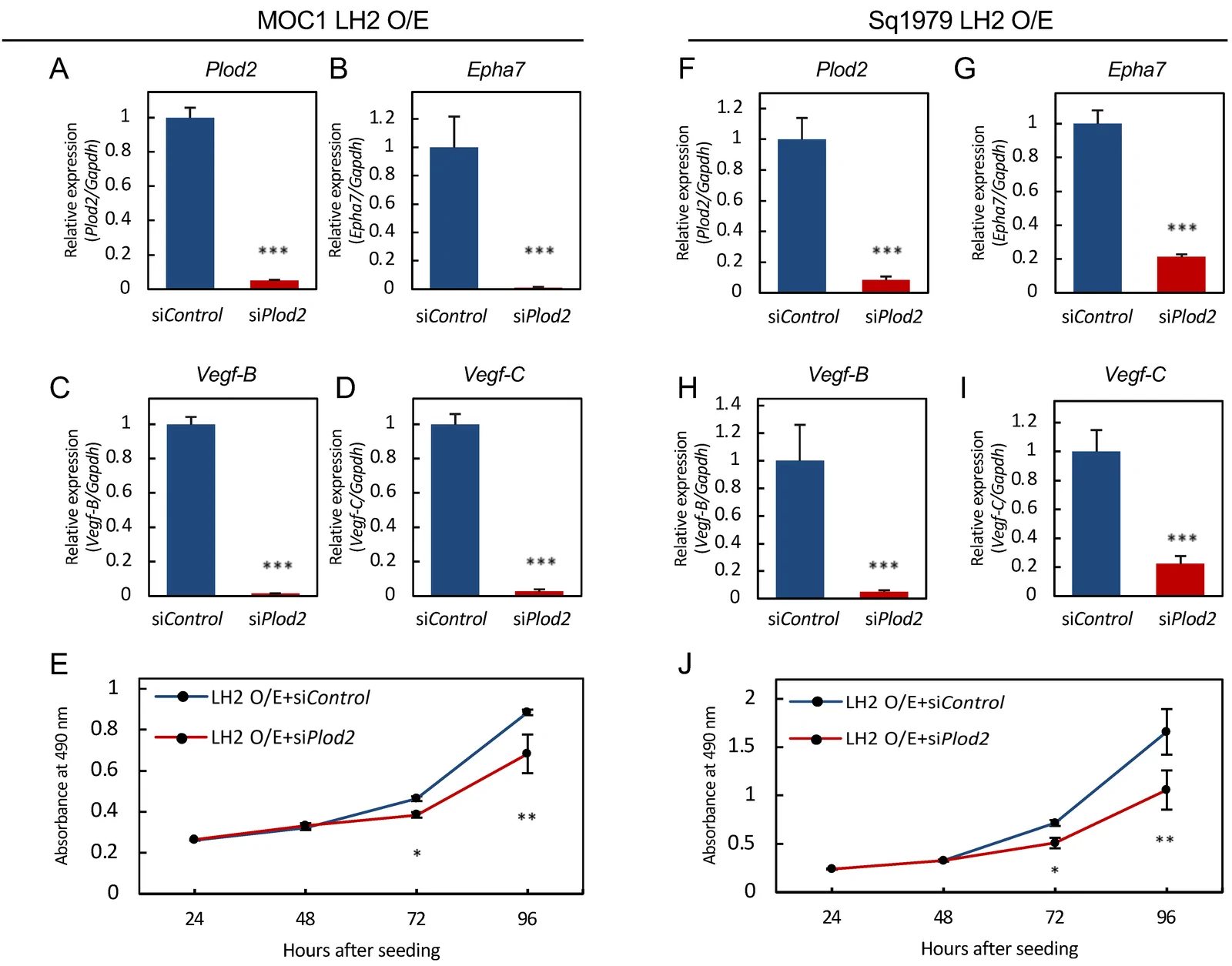
** Effects of *Plod2* knockdown in LH2 O/E OSCC cells.** (A, F) qRT-PCR analysis showing reduced *Plod2* mRNA expression in LH2 O/E MOC1 cells (A) and Sq1979 cells (F) transfected with si*Plod2* compared with those transfected with si*Control*. Relative *Plod2* mRNA levels normalized to *Gapdh* are shown (n = 3 per group; ***P < 0.001; Student's t-test). (B, G) qRT-PCR analysis showing reduced *Epha7* mRNA expression in LH2 O/E MOC1 cells (B) and Sq1979 cells (G) transfected with si*Plod2* compared with those transfected with si*Control* (n = 3 per group; ***P < 0.001; Student's t-test). (C, H) qRT-PCR analysis showing reduced *Vegf-B* mRNA expression in LH2 O/E MOC1 cells (C) and Sq1979 cells (H) transfected with si*Plod2* compared with those transfected with si*Control* (n = 3 per group; ***P < 0.001; Student's t-test). (D, I) qRT-PCR analysis showing reduced *Vegf-C* mRNA expression in LH2 O/E MOC1 cells (D) and Sq1979 cells (I) transfected with si*Plod2* compared with those transfected with si*Control* (n = 3 per group; ***P < 0.001; Student's t-test). (E, J) MTS assay showing significantly reduced cell proliferation in LH2 O/E MOC1 cells (E) and Sq1979 cells (J) transfected with si*Plod2* compared with those transfected with si*Control*. The si*Plod2*-transfected cells exhibited significantly reduced proliferation at 72 h (*P < 0.05) and 96 h (**P < 0.01). Data are presented as the mean ± SD from three independent experiments.

**Figure 6:**
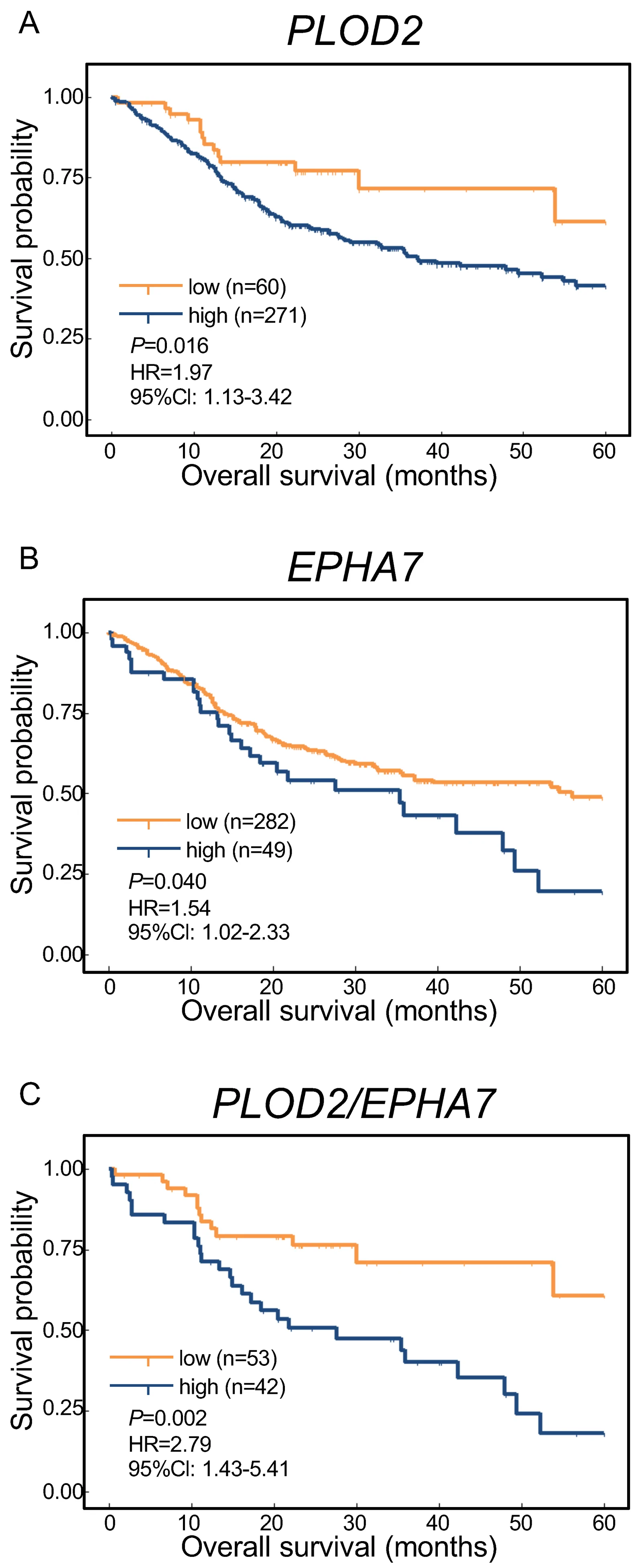
** Clinical relevance of *PLOD2* and *EPHA7* expression in the TCGA cohort.** Optimal cutoff values for *PLOD2* and *EPHA7* expression were determined separately using receiver operating characteristic (ROC) curve analysis based on their association with OS. The resulting cutoff values were 692.84 for *PLOD2* and 31.80 for *EPHA7*. Patients with expression values above the respective cutoff were classified as the high-expression group, whereas those with expression values below the cutoff were classified as the low-expression group. (A) For *PLOD2*, 271 patients were classified as high-expression and 60 as low-expression; (B) for *EPHA7*, 49 patients were classified as high-expression and 282 as low-expression. (C) For the combined analysis, patients were stratified according to the expression status of both genes. Patients with high expression of both *PLOD2* and *EPHA7* (*PLOD2*-high/EPHA7-high, n = 42) were compared with those with low expression of both genes (*PLOD2*-low/*EPHA7*-low, n = 53). Patients with discordant expression patterns (*PLOD2*-high/*EPHA7*-low or *PLOD2*-low/*EPHA7*-high) were excluded from the combined analysis.

**Figure 7:**
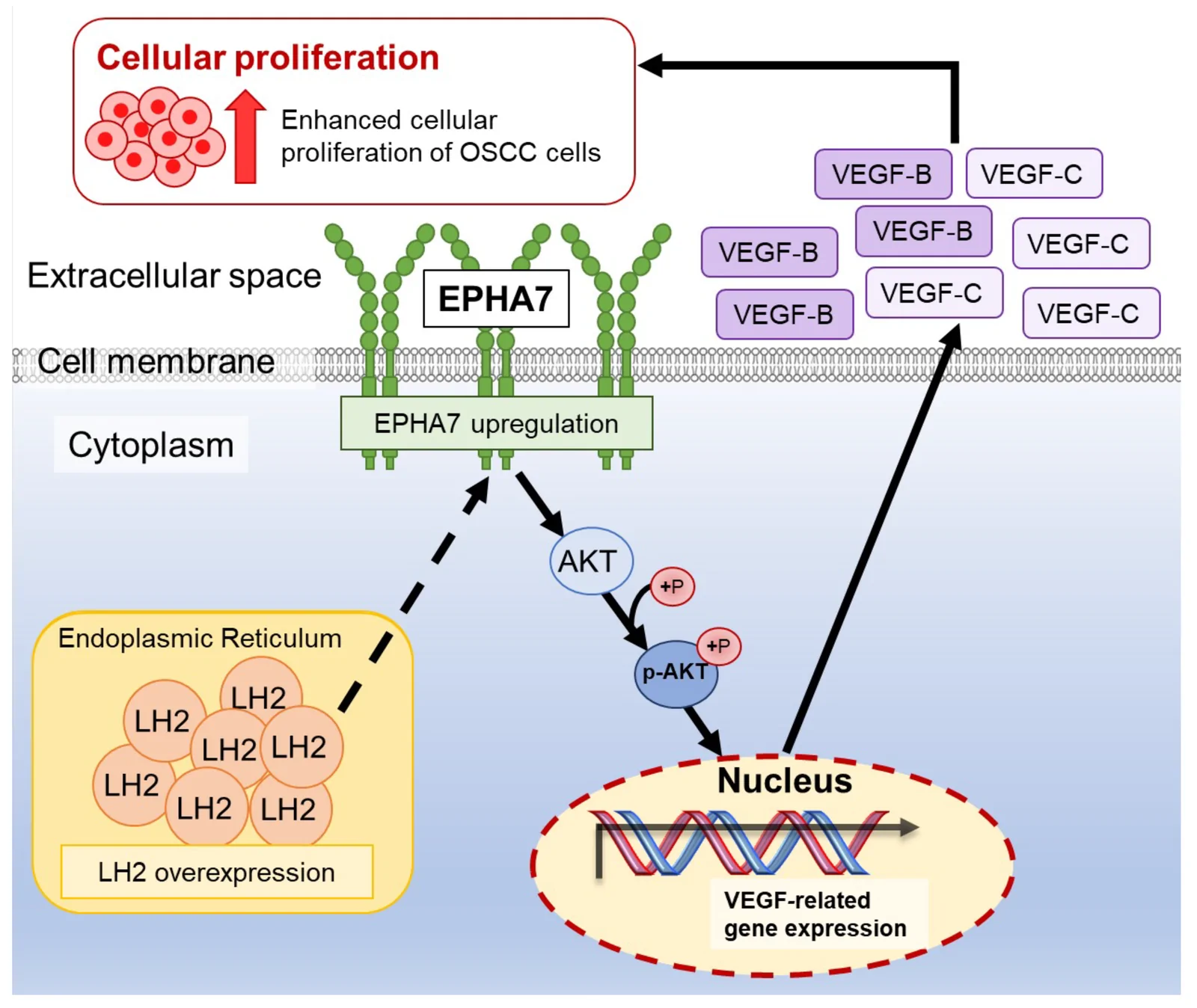
** Proposed model of the LH2-EPHA7-AKT-VEGF signaling axis in OSCC cells.** Schematic illustration of the proposed molecular events associated with LH2 overexpression in OSCC cells. LH2 is localized in the endoplasmic reticulum and is proposed to indirectly influence EPHA7 expression. Increased EPHA7 expression is associated with activation of AKT signaling through phosphorylation, accompanied by increased expression and secretion of VEGF-B and VEGF-C. These molecular events are associated with enhanced cellular proliferation in OSCC cells. The dashed arrow from LH2 to EPHA7 indicates a proposed indirect relationship rather than direct regulation. P, phosphorylation; LH2 O/E, LH2 overexpression.
