# Supplementary material for: LH2 Promotes Tumor Progression in Oral Squamous Cell Carcinoma via EPHA7-AKT-VEGF Signaling
Source: J Cancer. 2026 Sep 28;17(11):1829–40. doi: 10.7150/jca.141338 (PMC13686774; doi:10.7150/jca.141338)
Supplement: Supplementary file 1 [file jcav17p1829s1.pdf]

Supplementary information

LH2 Promotes Tumor Progression in Oral Squamous Cell Carcinoma via EPHA7–AKT–VEGF Signaling

Yuki Nishimura[1], Atsushi Kasamatsu[1,2]*, Reo Fukushima[2], Tomoaki Saito[1], Suguru Okoshi[1], Isao Miyamoto[2], Dai Nakashima[2], Katsuhiro Uzawa[1,2]*

[1]Department of Oral Science, Graduate School of Medicine, Chiba University, Chiba, Japan

[2]Department of Dentistry and Oral-Maxillofacial Surgery, Chiba University Hospital, Chiba, Japan

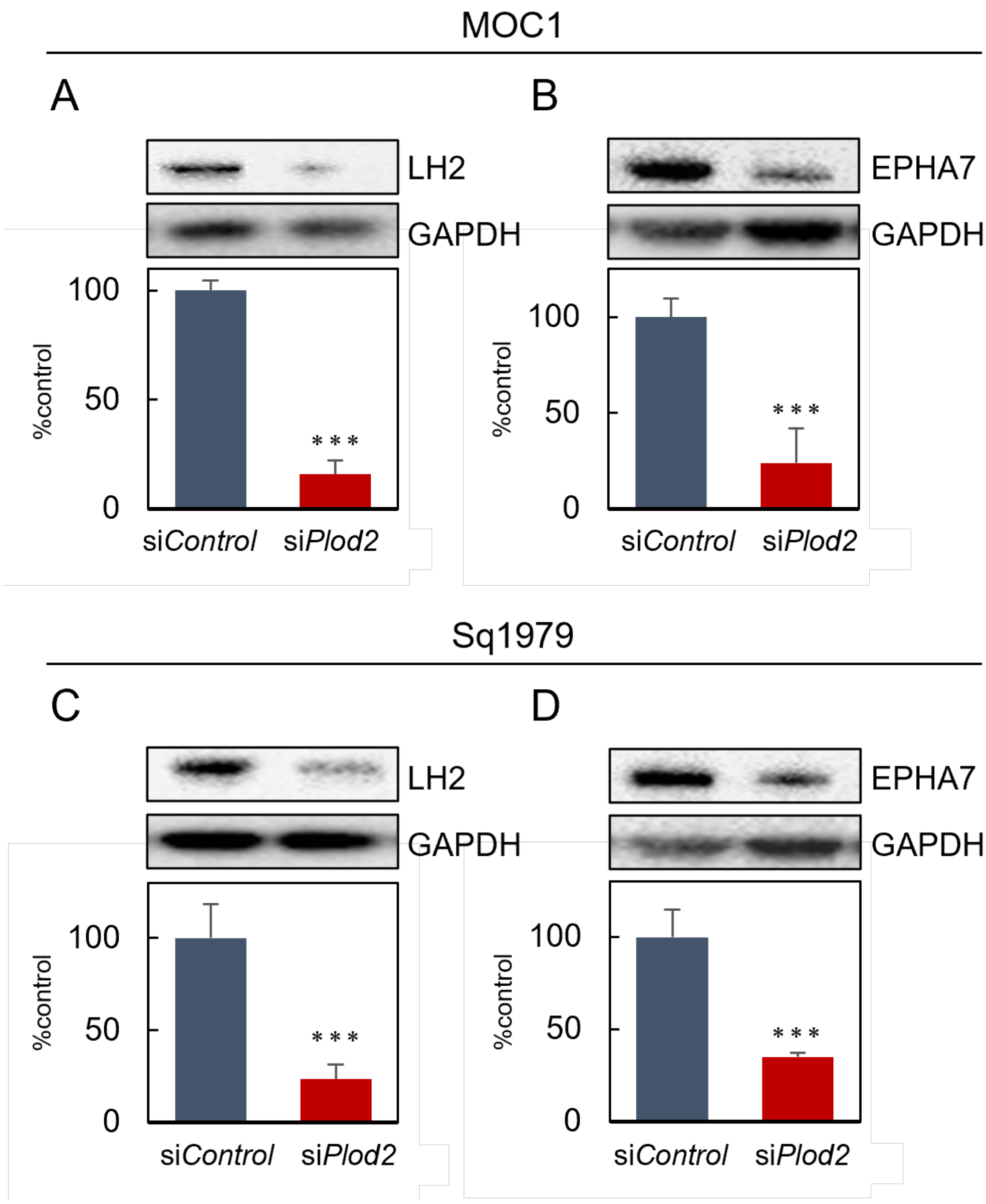

Supplementary Figure S1. Immunoblot analyses of LH2 and EPHA7 in si*Control* and si*Plod2* cells.

LH2 (A and C) and EPHA7 (B and D) protein expressions in si*Control* and si*Plod2* of LH2 O/E OSCC cells were determined by immunoblot analysis. Data are presented as mean ± SD (n = 3 per group).

Immunoblot analyses showed that LH2 and EPHA7 protein expressions were significantly (***P<0.001) decreased in si*Plod2* of LH2 O/E cells compared with si*Control*.
